# Ultra-uniform perovskite crystals formed in the presence of tetrabutylammonium bistriflimide afford efficient and stable perovskite solar cells†

**DOI:** 10.1039/d4ee01841a

**Published:** 2024-09-25

**Authors:** Jaekeun Lim, Alwani Imanah Rafieh, Naoyuki Shibayama, Jianxing Xia, Jean-Nicolas Audinot, Tom Wirtz, Sachin Kinge, Stefan W. Glunz, Yong Ding, Bin Ding, Hobeom Kim, Michael Saliba, Zhaofu Fei, Paul J. Dyson, Mohammad Khaja Nazeeruddin, Hiroyuki Kanda

**Affiliations:** a Institute of Chemical Sciences and Engineering, Swiss Federal Institute of Technology Lausanne (EPFL) Lausanne CH-1015 Switzerland zhaofu.fei@epfl.ch paul.dyson@epfl.ch mdkhaja.nazeeruddin@epfl.ch hiroyuki.kanda@epfl.ch; b Institute for Photovoltaics (ipv), University of Stuttgart Pfaffenwaldring 47 70569 Stuttgart Germany michael.saliba@ipv.uni-stuttgart.de; c Fraunhofer Institute for Solar Energy Systems ISE Heidenhofstr. 2 79110 Freiburg Germany; d Graduate School of Engineering, Toin University of Yokohama 1614 Kuroganecho, Aoba Yokohama Kanagawa 225-8503 Japan; e Advanced Instrumentation for Nano-Analytics (AINA), Luxembourg Institute of Science and Technology (LIST) L-4422 Belvaux Luxembourg; f Toyota Motor Europe, Toyota Motor Technical Centre, Advanced Technology Div. Hoge Wei 33 B-1930 Zaventem Belgium; g Department of Sustainable Systems Engineering (INATECH), University Freiburg Emmy-Noether-Str. 2 79110 Freiburg Germany; h School of Materials Science and Engineering, Gwangju Institute of Science and Technology (GIST) Gwangju 61005 Republic of Korea; i Helmholtz Young Investigator Group FRONTRUNNER, IEK5-Photovoltaik, Forschungszentrum Jülich Jülich Germany

## Abstract

Compositional engineering of organic–inorganic metal halide perovskite allows for improved optoelectrical properties, however, phase segregation occurs during crystal nucleation and limits perovskite solar cell device performance. Herein, we show that by applying tetrabutylammonium bistriflimide as an additive in the perovskite precursor solution, ultra-uniform perovskite crystals are obtained, which effectively increases device performance. As a result, power conversion efficiencies (PCEs) of 24.5% in a cell and 21.2% in a module are achieved, together with high stability under illumination, humidity and elevated thermal conditions.

## Abstract 2

We induced ultra-uniform perovskite crystals employing tetrabutylammonium bistriflimide additives in perovskite precursor solution, effectively increasing device efficiency and durability.

## Graphical abstract

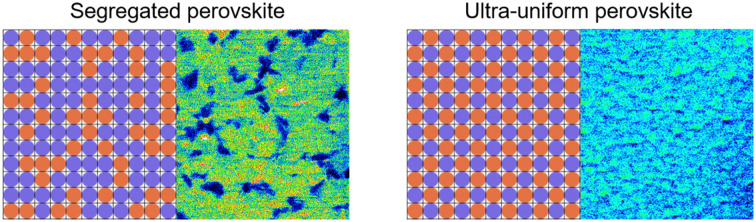

**Broader context**

Advancements in perovskite solar cell technology are crucial for enhancing their efficiency and stability, thereby making them more viable for commercial application. Here, we explore the innovative approach of ultra-uniform perovskites introduced by the tetrabutylammonium bistriflimide into the perovskite precursor solution. This method facilitates the formation of highly uniform crystals, thereby addressing the common issue of phase segregation that often occurs during the crystal nucleation process. By employing this technique, we have achieved notably high power conversion efficiencies (PCEs) of 24.5% in single cells and 21.2% in larger modules. Furthermore, these modified perovskite solar cells exhibit exceptional durability under challenging environmental conditions such as continuous light exposure, humidity, and elevated temperatures.

## Introduction

Perovskite solar cells (PSCs) have achieved power conversion efficiencies (PCEs) exceeding 26%, which is approaching the Shockley–Queisser limit.^1,2^ The efficiencies of PSCs have been improved by compositional engineering of perovskite absorbers,^3–7^ interfacial engineering of the layers in device structures,^8–12^ defect passivation^13–22^ and by encapsulating devices to reduce oxygen and moisture causing unwanted degradation of the perovskite layer.^23–25^ The long-term stability of PSCs is still a concern, and in order to commercialise PSCs, stability must be prolonged beyond the current state-of-the-art.

Early perovskite photovoltaics comprised mono-A-cation based materials. Mono-A-cation perovskites, *e.g.*, MAPbI_3_, FAPbI_3_ and CsPbI_3_, had issues involving phase transitions from the photoactive phase to the photo-inactive phase induced by light irradiation, moisture and high temperatures.^26–28^ Hence, mixed cation-based composition engineering was introduced to improve optoelectronic properties such as strong light absorption, long electron–hole diffusion length and low exciton binding energy, resulting in higher performance and stability.^29,30^ Mixed-cation composition engineering has advanced to multiple cation perovskites using various cations such as methylammonium (MA^+^), formamidinium (FA^+^), caesium (Cs^+^), rubidium (Rb^+^), potassium (K^+^) and sodium (Na^+^).^3,4,31,32^ However, these mixed cations systems encounter problems associated with phase segregation.^33^ Segregation induces inactive polycrystalline perovskites leading to defects in the grains and at the boundaries. Thus, phase segregation adversely affects film morphology, interfacial behaviour, carrier dynamics and device performance.^34^

Compositional uniformity is significantly important in the perovskite photovoltaic field for up-scaled perovskite modules beyond small-size perovskite solar cells, which are towards successful commercialization. Previous research has shown that uniform layers incredibly affect stability, which can retard degradation speed and afford better performance and reproducibility.^35–38^ Therefore, solving the phase segregation issue will lead to improved stability and reproducibility of devices.

We propose homogeneous perovskite crystals utilizing [N4444][TFSI], which strongly affects the photovoltaic performance of perovskite solar cells. The influence of homogeneous perovskite films was investigated by controlling the distribution of cations and anions in all crystal domains with varying concentrations of the additive. This compositional engineering finally provides ultra-uniform perovskite crystals that display high stability against light irradiation, thermal stress, and humidity.

## Results and discussion

**Fig. 1:**
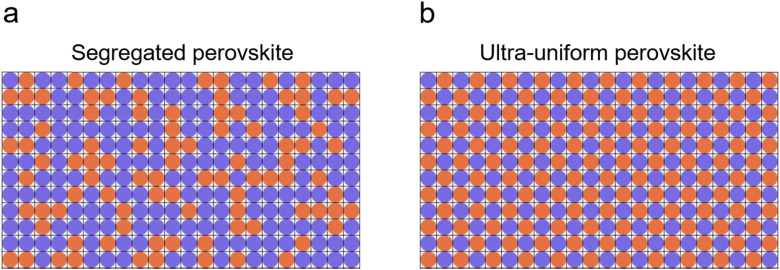
Schematics of segregated and ultra-uniform perovskite. (a) Segregated perovskite as a reference and (b) ultra-uniform perovskite as a target sample, representing uniformity of the cations/anions distribution. Blue and red circles correspond to cations and anions, respectively.

Schematics of segregated and ultra-uniform perovskites are shown in Fig. 1(a) and (b), respectively. Perovskite crystals controlled by adding tetrabutylammonium bistriflimide ([N4444][TFSI]) to the perovskite precursor solution (Fig. S1, ESI†) show more homogeneity in the cation/anion distribution. [N4444][TFSI] salt was added into the CsFAMAPbI_3_-based perovskite precursor solution to facilitate crystal growth. The [TFSI] anion potentially interacts with the metal ions as it is a weak Lewis base. Its O and/or N atoms are able to act as both monodentate and bidentate ligands, *i.e.* with the chelate effect, to form dative bonds with alkali metal ions such as Cs^+^ and Pb^2+^.^39^ It is reasonable to expect that this anion can interact with lead ions (Pb^2+^) in the perovskite precursor solution and the resulting perovskite.^40,41^ Thus, [N4444][TFSI] is expected to play a role as a nucleation modifier for the perovskite crystal.

**Fig. 2:**
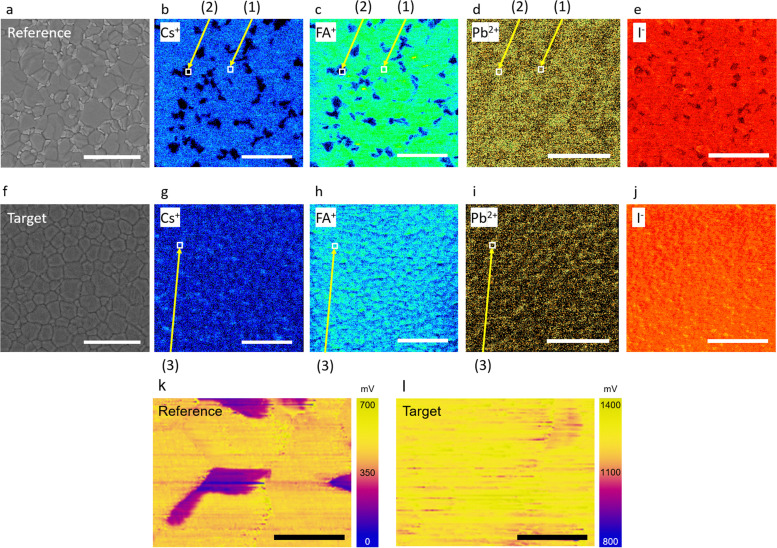
HIM-SIMS and KPFM mapping of perovskite layers. Reference sample: (a) secondary electron image, SIMS mapping for (b) Cs^+^, (c) FA^+^, (d) Pb^2+^, and (e) I^−^. Target sample: (f) secondary electron image, SIMS mapping for (g) Cs^+^, (h) FA^+^, (i) Pb^2+^, and (j) I^−^, scale bar is 5 μm. Surface potential of perovskite surface from KPFM for (k) reference and (l) target, scale bar is 1 μm.

The effects of [N4444][TFSI] on the nanometer-scale atomic distribution in perovskite films were investigated using Helium ion microscopy combined with secondary ion mass spectrometry (HIM-SIMS), as shown in Fig. 2. Fig. 2(a)–(e) show secondary electron (SE) images and HIM-SIMS images of Cs^+^, FA^+^, Pb^2+^ and I^−^ for the reference perovskite films, *i.e.* without [N4444][TFSI], and Fig. 2(f)–(j) show the target perovskite film. For SE, HIM-SIMS and KPFM mapping, the device stack of reference and target were (FTO/c-TiO_2_/mp-TiO_2_/CsFAPbI_3_) and (FTO/c-TiO_2_/mp-TiO_2_/CsFAPbI_3_ added with 5 mM of [N4444][TFSI]), respectively. In Fig. 2(a), the surface image of the reference sample displays SE image contrasts in perovskite grains as segregation, which may indicate compositional differences within the perovskite film. HIM-SIMS images of the reference sample reveal that Cs^+^, FA^+^, and I^−^ are segregated (Fig. 2(b)–(e)), which is consistent with the SEM images (Fig. S2–S4 and Table S1, ESI†). The presence of small and segregated grains can result in high recombination losses and limits the photovoltaic performances.^33,42^ Notably, the addition of [N4444][TFSI] to the perovskite precursor solution substantially suppresses segregation, as shown in Fig. 2(f)–(j). The Cs^+^ and FA^+^ (Fig. 2(g) and (h)) and I^−^ (Fig. 2(j)) are distributed more uniformly compared to the reference film (Fig. 2(b), (c) and (e)). These results suggest that the uniform perovskite crystals induced by the addition of [N4444][TFSI] in the target sample may suppress recombination sites and improve the photovoltaic performance of the target device. In Fig. 2(b)–(d), the numbers (1) and (2) indicate the positions of the non-segregated and segregated parts in the reference, respectively. Mole fractions shown in Table 1 are normalized by the mole fraction of reference indicated by position (1). The number of mole fractions in Table 1 shows that the mole fractions of the Cs^+^ (0.003), FA^+^ (0.080) and Pb^2+^ (0.916) ions at position (2) are different from the position (1) (Cs^+^ (0.025), FA^+^ (0.475), and Pb^2+^ (0.500)). This is due to the segregation of cations and anions at the segregated part at position (2) with respect to the non-segregated part of the same reference at position (1). Interestingly, in the target sample, the mole fractions of Cs^+^ (0.033), FA^+^ (0.431), and Pb^2+^ (0.536) at position (3) are similar to that of the reference at position (1) Cs^+^ (0.025), FA^+^ (0.475), and Pb^2+^ (0.500). This suggests that the [N4444][TFSI] plays a role in inducing the highly uniform perovskite crystal without segregation. Moreover, Kelvin probe force microscope (KPFM) was used to map the surface potential of the perovskite films (Fig. 2(k) and (l)). The reference sample (Fig. 2(k)) shows that there are partially low potential (<350 mV) domains in the perovskite film, which might be due to the segregation of the Cs^+^, FA^+^ and I^−^ in the perovskite grain (Fig. 2(b), (c) and (e)). In contrast, the surface potential of the target film with [N4444][TFSI] (Fig. 2(l)) is considerably more uniform with higher potential than the reference, which is consistent with the SIMS results (Fig. 2(g)–(j)). Hence, we found that [N4444][TFSI] promotes the formation of a high and uniform surface potential.^43,44^ During the nucleation process, the concentration of the [N4444][TFSI] in the perovskite solution will increase significantly towards the end and it can form eutectic ionic liquids with perovskite precursors, where several cations including [N4444]^+^, Cs^+^, FA^+^, Pb^2+^, and anions including [TFSI]^−^ and I^−^ can co-exist. Unlike DMSO which is a volatile solvent, the [N4444][TFSI] will not be evaporated due to its ionic nature. It will stay throughout the entire process and accumulate at the surface after the completion of the nucleation process.^45^

**Table 1:** Mole fractions of each perovskite grain for perovskite cations

Position	Remarks	Cs^+^	FA^+^	Pb^2+^
(1)	Non-segregated part in reference	0.025	0.475	0.500
(2)	Segregated part in reference	0.003	0.080	0.916
(3)	Target	0.033	0.431	0.536

**Fig. 3:**
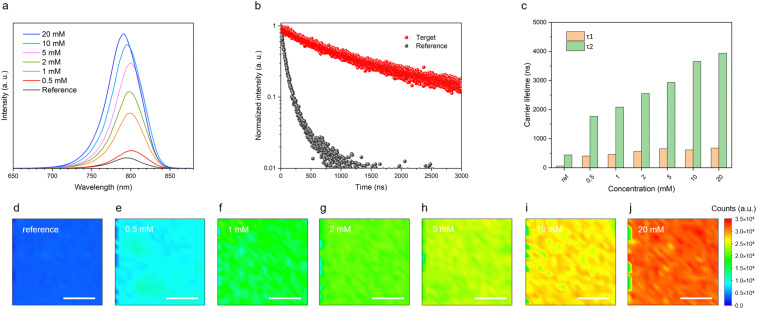
Photoluminescence spectroscopy of the perovskite films. (a) Steady-state photoluminescence emission (PL emission) spectra, (b) time-resolved photoluminescence (tr-PL) of the reference (without [N4444][TFSI]) and target (5 mM of [N4444][TFSI]), (c) carrier lifetime, and (d)–(j) photoluminescence mapping (PL mapping) of the perovskite films with 0, 0.5, 1, 2, 5, 10, and 20 mM concentrations of [N4444][TFSI] in the perovskite precursor solution. Scale bar is 200 μm.

Steady-state photoluminescence emission (PL emission), time-resolved photoluminescence (tr-PL) spectroscopy, and photoluminescence mapping (PL mapping) were used to characterize the perovskite films (FTO/c-TiO_2_/mp-TiO_2_/CsFAPbI_3_) employing different concentrations of [N4444][TFSI], from 0 to 20 mM, added to the perovskite precursor solution. The main peak of the PL emission is close to 800 nm, which intensifies with increasing concentrations of [N4444][TFSI] in the perovskite layer, suggesting that [N4444][TFSI] reduces nonradiative pathways in Fig. 3(a).^46–48^ This observation is consistent with the enhanced emission lifetime shown in Fig. 3(b), (c) and Table S2 (ESI†), indicating that the increased *τ*_1_ and *τ*_2_ values may be attributed to the suppression of band-to-band recombination^49,50^ (note that the perovskite thickness is the same for all the samples). Moreover, the PL mapping image of the reference film shows a uniform photoluminescence intensity in all the films containing [N4444][TFSI] (Fig. 3(d)–(j)). The absolute emission intensity gradually increases with the [N4444][TFSI] concentration, *i.e.* the [N4444][TFSI] added perovskite improves photoelectronic properties, presumably leading to the improved photovoltaic performance of perovskite solar cells (see below).

**Fig. 4:**
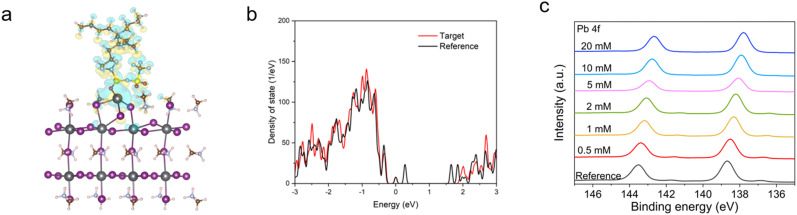
Calculated tDOS (total density of state) and analysis of the perovskite surfaces. (a) Charge density difference of the [N4444][TFSI]-passivated perovskite surface and (b) tDOS of [N4444][TFSI]-passivated (red line) and non-passivated (black line) perovskite surface. (c) Pb 4f XPS spectra with various concentrations of [N4444][TFSI].

X-ray diffraction (XRD) and X-ray photoelectron spectroscopy (XPS) were characterized with the perovskite films (FTO/c-TiO_2_/mp-TiO_2_/CsFAPbI_3_) employing different concentrations of [N4444][TFSI], from 0 to 20 mM, added to the perovskite precursor solution. For the grazing incidence wide-angle X-ray scattering (2D-GIWAX) and SEM measurement, the device stack of reference and target were prepared as (FTO/c-TiO_2_/mp-TiO_2_/CsFAPbI_3_) and (FTO/c-TiO_2_/mp-TiO_2_/CsFAPbI_3_ added with 5 mM of [N4444][TFSI]), respectively. XRD and 2D-GIWAXS show that [N4444][TFSI] does not alter the orientation of perovskite crystal lattice as there is no shift in (100) peak, see Fig. S5–S7 (ESI†). The addition of [N4444][TFSI], however, affects the morphology of perovskite film, with the average crystallite size increased compared to the reference film, see Fig. S3 (ESI†). The average crystallite size of perovskite with 1 mM of [N4444][TFSI] is 0.888 μm^2^, which is higher than that of the reference film (0.684 μm^2^). To investigate the interaction between [N4444][TFSI] and the perovskite grains, density functional theory (DFT) calculations were undertaken and X-ray photoelectron spectroscopy (XPS) was recorded, as shown in Fig. 4. Fig. 4(a) shows the charge density difference of [N4444][TFSI] adsorbed on the Pb^2+^ terminated (001) perovskite surface. The electronic coupling of [N4444][TFSI] on the perovskite is depicted visually in an isosurface plot of the charge density difference (Δ*ρ*). In the PbI_2_ terminated model, a strong electron depletion (Δ*ρ* < 0, negative) appearing around the undercoordinated Pb^2+^ defect on the perovskite surface is inverse to the electron accumulation (Δ*ρ* > 0, positive) around the [N4444][TFSI] where it is in contact with the perovskite surface. This implies that [TFSI]^−^ can coordinate with Pb^2+^ defect on the perovskite surface, *i.e.* passivating the surface of the perovskite film.^51^ To determine the passivation effect of [N4444][TFSI] on the perovskite surface, density of states (DOS) of the perovskite was further studied (Fig. 4(b)), by comparing [N4444][TFSI]-passivated and non-passivated (black line) perovskite films. Trap states were initially generated within the band gap of the perovskite when Pb^2+^ defects are present on the perovskite surface, however the trap states sharply decrease when the [TFSI]^−^ anions bind to the defect-sites. XPS and ultraviolet photoelectron spectroscopy (UPS) analysis were performed to scrutinize the interaction between [N4444][TFSI] and defects of the perovskite crystal structure and to investigate the energy band diagram, respectively (Fig. 4(c), Fig. S8–S11 and Table S3, ESI†). The reference perovskite and [N4444][TFSI] added perovskite show different values of highest occupied molecular orbital (HOMO) and lowest unoccupied molecular orbital (LUMO) (−6.12 and −4.56 eV for reference; −5.99 and −4.45 eV for perovskite film with additives). The reason why [N4444][TFSI] changed energy level might be the change of the surface termination by ionic anchoring.^52,53^ Furthermore, target sample (FTO/c-TiO_2_/meso-TiO_2_/CsFAPbI_3_ with 1 mM [N4444][TFSI]) was measured by Time of Flight Secondary Ion Mass Spectrometry (ToF-SIMS) depth profiling to clarify the location and state of residual [N4444][TFSI] in the perovskite thin film after contributing to the compositional uniformity during crystallization. Fig. S13 (ESI†) clearly shows that most of the ionic liquid is distributed on the surface of the perovskite film. For more details, [N4444]^+^ is located on the top surface of the perovskite film. [TFSI]^−^ is located on the top surface of the perovskite film and the interface between TiO_2_ and perovskite film. There is a shift of the Pb 4f peak from 138.7 to 137.8 eV as the concentration of [N4444][TFSI] increases, indicative of an interaction between the Pb^2+^ ions and [N4444][TFSI]. The XPS of the reference film contains Pb 4f_5/2_ and Pb 4f_7/2_ peaks at 141.4 and 137 eV,^54,55^ respectively, due to the presence of metallic lead, which is not observed for the [N4444][TFSI]-added perovskites, consistent with the calculations of the [N4444][TFSI]-passivated perovskite surface (Fig. 4(a) and (b)). Additionally, Fourier transform infrared spectroscopy (FTIR) of perovskite films with varying amounts (from 0 to 100 mM) of [N4444][TFSI] in perovskite precursor solution indicates that the additive also may interact with the FA^+^ ion, demonstrated by the C–H bending peak of at 1352 cm^−1^ shown Fig. S12 (ESI†).^56,57^

**Fig. 5:**
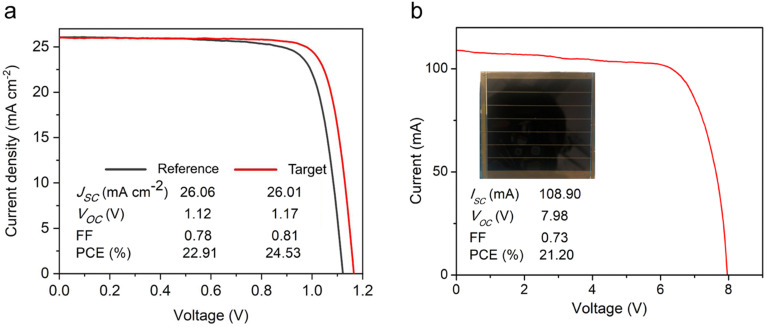
Photovoltaic performance of perovskite solar cells and the best performing target module. (a) Photovoltaic parameters including short circuit current density (*J*_sc_), open circuit voltage (*V*_oc_), fill factor (FF), and power conversion efficiency (PCE) of the reference (black curved line) and the target (red curved line) perovskite solar cells, and (b) target (red curved line) module of best performing perovskite solar cell with [N4444][TFSI] (inset contains a photograph of the module).

PSC devices were fabricated with the following structure, (FTO/TiO_2_/perovskite/*n*-octylammonium iodide (OAI)/spiro-MeOTAD/Au), and their photovoltaic properties assessed. The PCE of the reference device and the best-performing device, *i.e.* with 1 mM [N4444][TFSI], are 22.91 and 24.53%, respectively, see Fig. 5(a), attributed to the significant increase in *V*_OC_ (1.17 V) compared to that of the reference device (1.12 V) and superior photovoltaic properties (Fig. S14–S21, ESI†). In addition, the FF of the target device is 81%, which is higher than that of the reference FF of 78%. The improvement in the FF may be correlated to improved charge carrier transport, which indicates that the trap defects are reduced in the target device (Fig. S15, ESI†) due to the uniform perovskite layer and also the passivation effect.^58^ Some of target devices’ performance is relatively lower than the reference. It might originate from the compact TiO_2_ deposition by a spray coating method, which can induce pin-hole on the substrate due to ambient dust for instance. UPS and absorption characteristics show that the [N4444][TFSI] almost did not change the bandgap. However, the valence band maximum and conduction band minimum of the perovskite were elevated by the [N4444][TFSI] & OAI passivation, which can diminish the energy barrier and minimize the loss for hole transfer from perovskite to spiro-MeOTAD *via* the OAI passivation layer (Fig. S11, ESI†). Moreover, the elevated conduction band by a quasi-2D perovskite layer with OAI can block the electrons to spiro-MeOTAD effectively, which reduces the interface recombination and improves open circuit voltage and fill factor. Although the SEM shows a similar thickness of perovskite film, the average of *J*_sc_ increased by the [N4444][TFSI], possibly due to the suppression of the defects for the recombination center, which is consistent with the photoluminescence result (Fig. 3). To investigate the residual effect of the [N4444][TFSI] on device performance, we tried to deposit [N4444][TFSI] on the perovskite layer, which associates with the residual [N4444][TFSI] (Fig. S19, ESI†). The device structure is (FTO/ETL/perovskite without additives/[N4444][TFSI] passivation layer/OAI passivation layer/HTM/Au). The highest efficiency of the device with residual [N4444][TFSI] & OAI was higher than the reference but lower than the target device. This indicates that the residual [N4444][TFSI] can play a role in surface passivation and improve photovoltaic performance. This result suggests that the effect of the residual [N4444][TFSI] and compositional uniformity is different and they can synergistically increase the device performance of the perovskite solar cells. We also confirmed the effect of the OAI passivation on the perovskite solar cells, which improved photovoltaic performance (Fig. S19, ESI†). This result indicates that the advantages of passivation and uniformity stand independently, improving the device performance of the perovskite solar cells with the different approaches. Moreover, we demonstrated with bromine doped perovskite solar cells (≈1.6 eV) and p–i–n stack (Fig. S15 and S16, ESI†). These photovoltaic performances were increased by the [N4444][TFSI], which is consistent with the improvement of the parameters shown in Fig. 5. These results can support the generality of the approach. A module with an active area of 30 cm^2^ was also fabricated with a PCE of 21.2% obtained for the target module, Fig. 5(b) shows the *J*–*V* characteristics of the module consisting of seven cells connected in series.

**Fig. 6:**
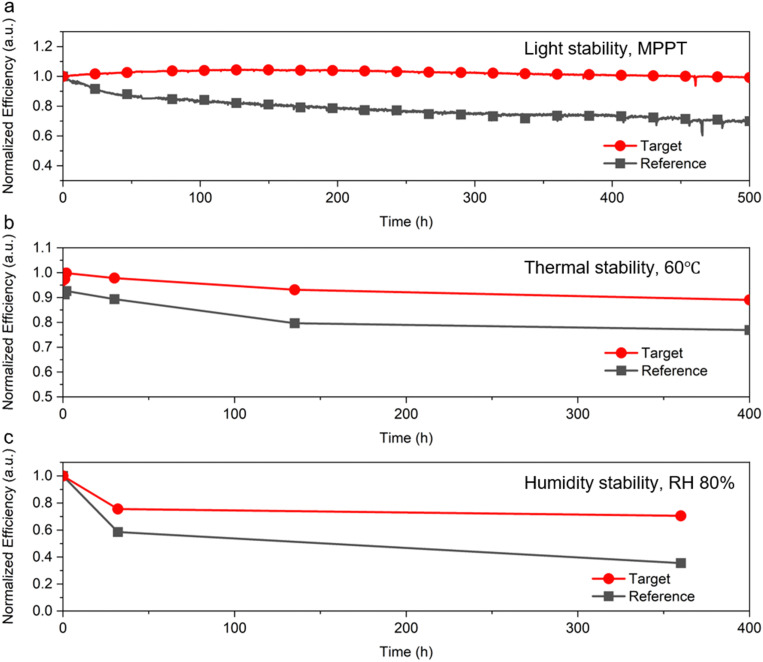
Stability of perovskite solar cells. (a) Maximum-power point tracking under 1 sun irradiation, (b) thermal stability at 60 °C, and (c) humidity stability under 80% relative humidity.

The target devices showed improved stability, retaining almost 100% of the initial PCE after 500 hours of maximum-power point tracking (MPPT) under 1 sun irradiation, compared to that of the reference devices which retained 70% of their initial PCE (Fig. 6(a)). Moreover, the [N4444][TFSI]-added perovskite results in improved stability to heat and relative humidity (RH) (Fig. 6(b) and (c)). Under a thermal condition of 60 °C, the target device retained 90% of the initial PCE after 400 hours, and the target device retained 70% of initial PCE after 360 hours under relative humidity of 80%. Statistics data for stability tests are shown in Fig. S20 (ESI†). Number of samples was three for thermal stability and humidity stability tests. Regarding thermal stability (60 °C), the target sample maintained an average of 84% from the initial efficiency, which was higher than that of devices (72%). As for humidity stability (80% RH), target and reference samples maintained an average of 67% and 40% from the initial efficiency, respectively. The highly hydrophobic nature of the [N4444][TFSI] accumulated at the interface helps to prevent hydrolysis against moisture. Indeed, [N4444][TFSI] is highly hydrophobic and the contact angle of target film is 73.6°, with the reference perovskite film contact angle being only 67.0° (Fig. S22, ESI†). Overall, these results indicated that the uniformity of the perovskite crystal and hydrophobic additives result in improving the stability of the perovskite solar cells.

## Conclusions

We prepared ultra-uniform perovskite absorber films using [N4444][TFSI] as an additive in the perovskite precursor solution. The resulting perovskite films show no signs of elemental segregation and their highly uniform nature improves photovoltaic properties when applied in perovskite solar cells. We found that the uniformity of the perovskite helps to achieve highly stable perovskite solar cells under illuminating, thermal and humid conditions. Our findings provide an approach to suppress segregated perovskite grains.

## Experimental

### Materials

Fluorine-doped tin oxide (FTO) glasses were purchased from Asahi Glass. [N4444][TFSI] was synthesised using a modified reference protocol.^59^ Tetrabutylammonium bromide, methylammonium chloride (Sigma Aldrich), *n*-octylammonium iodide (Greatcell Solar), lead(ii) iodide, [6,6]-phenyl-C_61_-butyric acid methyl ester, and bathocuproine (Xi’an Polymer Light Technology Corp.), formamidinium iodide (Xi’an Polymer Light Technology Corp.), cesium iodide (ABCR, GmbH), dimethylformamide and ethanol (Sigma Aldrich), dimethyl sulfoxide (Sigma Aldrich), titanium dioxide paste and phenethylammonium iodide (GreatCell Solar), ethanol (Fisher), isopropanol (Acros Organics), diethyl ether (Acros Organics), titanium diisopropoxide bis(acetylacetonate) (Sigma Aldrich), chlorobenzene (Sigma Aldrich), spiro-OMeTAD (2,2′,7,7′-tetrakis[*N*,*N*-di(4-methoxyphenyl)amino]-9,9′-spirobifluorene) (Luminescence technology corp.), lithium bistriflimide (GreatCell Solar), FK 209 cobalt(iii) bistriflimide (GreatCell Solar), acetonitrile (Acros Organics), 4-*tert*-butylpyridine (Sigma Aldrich), were all used without any further purification.

### Synthesis of tetrabutylammonium bistriflimide ([N4444][TFSI])

Lithium bistriflimide (0.287 g, 1.0 mmol, 1.0 equiv.) and tetrabutylammonium bromide (0.322 g, 1.0 mmol, 1.0 equiv.) was mixed in H_2_O (10 mL). The mixture was stirred at room temperature overnight, after which it was filtered and the solid was collected by filtration followed by washing with water (4 × 10 mL). [N4444][TFSI] was dried and recrystallised from acetone. Yield: 501 mg (96%).

### Device fabrication

The fluorine-doped tin oxide (FTO) glasses were cleaned with detergent, deionised (DI) water, and 10% volume of hydrochloric acid (HCl) with DI water, acetone and isopropanol using an ultrasonic bath for 20 min as a sequential process. The FTO substrates were treated by UV/ozone cleaner for 15 minutes before depositing the compact titanium dioxide (c-TiO_2_). The c-TiO_2_ film was deposited on the FTO substrates using spray pyrolysis with a titanium diisopropoxide bis(acetylacetonate) solution diluted in ethanol at a 1 : 20 volume ratio. This deposition process was conducted *in situ* annealing for 1 hour on a hotplate at 450 °C. The FTO/c-TiO_2_ substrates were treated with UV/ozone and was then cooled to room temperature for 15 min. The meso porous titanium dioxide (mp-TiO_2_) solution was prepared from 1 g of TiO_2_ paste diluted in 10 mL of ethanol solvent. The mp-TiO_2_ film was prepared by spin coating at 3000 rpm for 30 s. The mp-TiO_2_ films deposited on FTO/c-TiO_2_ substrates were annealed at 500 °C in air. The perovskite precursor solutions were prepared by dissolving 1.3 M FAPbI_3_ with CsI (10 mM%) and MACl (30 mol%) in mixed solvent of dimethylformamide : dimethyl sulfoxide = 4 : 1 (control); 1 mM of [N4444][TFSI] was added (Target). The perovskite solutions were spin-coated onto the substrate at 1000 rpm for 10 s and 5000 rpm for 20 s; 500 ml of ethyl ether was dripped onto the substrate during spinning. Heat-treatment was implemented with the substrates for 15 min at 150 °C. Next, 8 mg of *n*-octylammonium iodide in 1 mL of isopropanol (IPA) was deposited onto the substrates using spin coating at 4000 rpm for 10 s and annealed at 100 °C. The hole-transport layer was prepared with 80 mg of spiro-OMeTAD solution in 1023 μL of chlorobenzene, sequentially adding 32 μL of 4-*tert*-butylpyridine, 19 μL of lithium bistriflimide salt solution (517 mg mL^−1^) in acetonitrile, and 14 μL of cobalt-complex solution (376 mg mL^−1^) in acetonitrile. The prepared spiro-OMeTAD solution was deposited onto the substrate at 4000 rpm for 30 s. 80 nm of the gold electrode was evaporated using thermal evaporation. For inverted structure device, patterned indium tin oxide (ITO) glasses were cleaned with the same procedure above. ITO substrates were treated by UV/ozone cleaner for 15 min. 2PACz of 0.35 mg in 1 mL of ethanol was deposited on ITO substrates at 4000 rpm for 30 s and annealed at 100 °C for 10 min. Perovskite absorber was deposited with same method above but annealed at 100 °C for 10 min. Next, 3 mg of PEAI in 1 mL IPA was deposited onto the substrates using 4000 rpm for 20 s. 20 mg of PCBM in CB was deposited with 2000 rpm for 30 s. 5 mg of BCP in IPA was deposited with 5000 rpm for 30 s. PEAI, PCBM and BCP were deposited with dynamic spin coating method. 100 nm of the silver electrode was evaporated using thermal evaporation.

## Characterisation

Structural secondary electron micrographs and chemical secondary ion mass spectrometry maps of the perovskite photon absorber were carried out at a beam energy of 15–20 keV on a HIM-SIMS instrument which is a helium ion microscope (HIM) (ORION NanoFab, Zeiss) coupled to a SIMS system developed by LIST.^60,61^ Time-resolved photoluminescence spectroscopy was performed using a Fluorolog TCSPC with an excitation wavelength of 640 nm (HORIBA, Ltd). PL mapping was performed on an inVia confocal Raman microscope (Renishaw plc.). X-ray photoelectron spectroscopy (XPS) was performed on a VersaProbe II (Physical Electronics, Inc.) with a monochromator and Al-Kα source of 1486.6 eV. Spectra were referenced using adventitious carbon. Fourier transform infrared (FTIR) spectroscopy was acquired using attenuated total reflectance (ATR) (Vertex-70V). *I*–*V* measurements were performed using an Oriel VeraSol solar simulator (Newport Corporation) and a LCE-50 (Centronics) calibrator, from 1.2 to 0 V and from 0 to 1.2 V as reverse and forward scans, respectively, using a mask of 0.0774 mm^2^. The scanning step and speed were 10 mV and 50 mV s^−1^, respectively. Maximum power tracking and stability tests were performed under 1 sun (100 mW cm^−2^) illumination in a nitrogen atmosphere with 0% humidity at 25 °C. SEM images were obtained using a field emission scanning electron microscope (FEI, Teneo SEM). UV/VIS spectra were recorded on a Lambda 950S instrument (PerkinElmer, Inc.). Photoluminescence emission spectra were obtained on a LS-55 fluorescence spectrometer (PerkinElmer, Inc.) with an excitation wavelength of 610 nm. 2D-GIWAXS images represented in the reciprocal lattice space were conducted at the BL19B2 beamline at SPring-8 (Japan). The perovskite films were irradiated with an X-ray energy of 12.39 keV at a fixed-incident angle on the order of 2.0 through a Huber diffractometer. The 2D-GIWAXS images were recorded with a two-dimensional image detector (Pilatus 300 K). The ultraviolet photoelectron spectroscopy (UPS) equipped with He–I source (*hν* = 21.22 eV) (AXIS Nova, Kratos Analytical Ltd, UK) was used to determine the valence band energy and Fermi-level.

## Author contributions

J. Lim, A. I. Rafieh, and H. Kanda fabricated devices, performed fundamental characterizations and wrote the manuscript. J. Lim and A. I. Rafieh have equal contributions as the first author. H. Kanda conceived the idea. N. Shibayama performed WAXS and GIWAXS measurements. J. Xia performed the DFT calculations. J.-N. Audinot and T. Wirtz performed ToF-SIMS. Y. Ding and B. Ding assisted with module fabrication. Z. Fei synthesized materials. H. Kanda, Z. Fei, M. Saliba, P. J. Dyson, and M. K. Nazeeruddin supervised the research. All authors contributed to discussions and to finalizing the manuscript.

## Data availability

All data supporting the findings of this study are included in the main text and the ESI.† All of relevant data are available from the corresponding authors upon reasonable request.

## Conflicts of interest

There are no conflicts to declare.

## Supplementary Material

EE-017-D4EE01841A-s001
