# Supplementary material for: Ultra-uniform perovskite crystals formed in the presence of tetrabutylammonium bistriflimide afford efficient and stable perovskite solar cells
Source: Energy Environ Sci. 2024 Sep 25;17(21):8209–18. doi: 10.1039/d4ee01841a (PMC11444314; doi:10.1039/d4ee01841a)
Supplement: EE-017-D4EE01841A-s001 [file EE-017-D4EE01841A-s001.pdf]

Supplementary Information (SI) for Energy & Environmental Science.
This journal is © The Royal Society of Chemistry 2024

Supplemental information

Ultra-uniform Perovskite Crystals Formed in the Presence of Tetrabutylammonium bistriflimide Afford Efficient and Stable Perovskite Solar Cells

Jaekeun Lim[†1,2,3], Alwani Imanah Rafieh[†1], Naoyuki Shibayama[4], Jianxing Xia[1], Jean-Nicolas Audinot[5], Tom Wirtz[5], Sachin Kinge[6], Stefan W. Glunz[3,7], Yong Ding[1], Bin Ding[1], Hobeom Kim[1,8], Michael Saliba[2,9]*, Zhaofu Fei[1]*, Paul J. Dyson[1]*, Mohammad Khaja Nazeeruddin[1]*, and Hiroyuki Kanda[1]*

[1] *Institute of Chemical Sciences and Engineering, Swiss Federal Institute of Technology Lausanne (EPFL), Lausanne CH-1015, Switzerland.*

[2] *Institute for Photovoltaics (ipv), University of Stuttgart, Pfaffenwaldring 47, 70569 Stuttgart, Germany.*

[3] *Fraunhofer Institute for Solar Energy Systems ISE, Heidenhofstr. 2, 79110 Freiburg, Germany.*

[4] *Graduate School of Engineering, Toin University of Yokohama, 1614 Kuroganecho, Aoba, Yokohama, Kanagawa, 225–8503 Japan*

[5] *Advanced Instrumentation for Ion Nano-Analytics (AINA), Materials Research and Technology Department, Luxembourg Institute of Science and Technology (LIST), L-4422 Belvaux, Luxembourg.*

[6] *Toyota Motor Corporation, Toyota Motor Technical Centre, Advanced Technology Div., Hoge Wei 33, B-1930 Zaventem, Belgium.*

[7] *Department of Sustainable Systems Engineering (INATECH), University Freiburg, Emmy-Noether-Str. 2, 79110 Freiburg, Germany.*

[8] *School of Materials Science and Engineering, Gwangju Institute of Science and Technology (GIST), Gwangju, 61005, Republic of Korea.*

[9] *Helmholtz Young Investigator Group FRONTRUNNER, IEK5-Photovoltaik, Forschungszentrum Jülich, Jülich, Germany.*

†Jaekeun Lim and Alwani Imanah Rafieh contributed equally to this work.

Corresponding authors: Michael Saliba, michael.saliba@ipv.uni-stuttgart.de; Zhaofu Fei, zhaofu.fei@epfl.ch; Paul J. Dyson, paul.dyson@epfl.ch; Mohammad Khaja Nazeeruddin, mdkhaja.nazeeruddin@epfl.ch; Hiroyuki Kanda, hiroyuki.kanda@epfl.ch

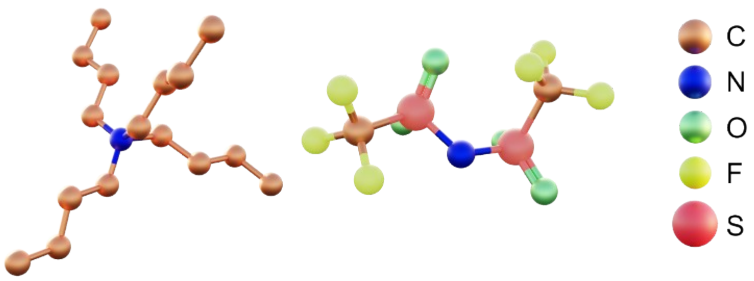

Fig. S1. Structure of [N4444][TFSI].

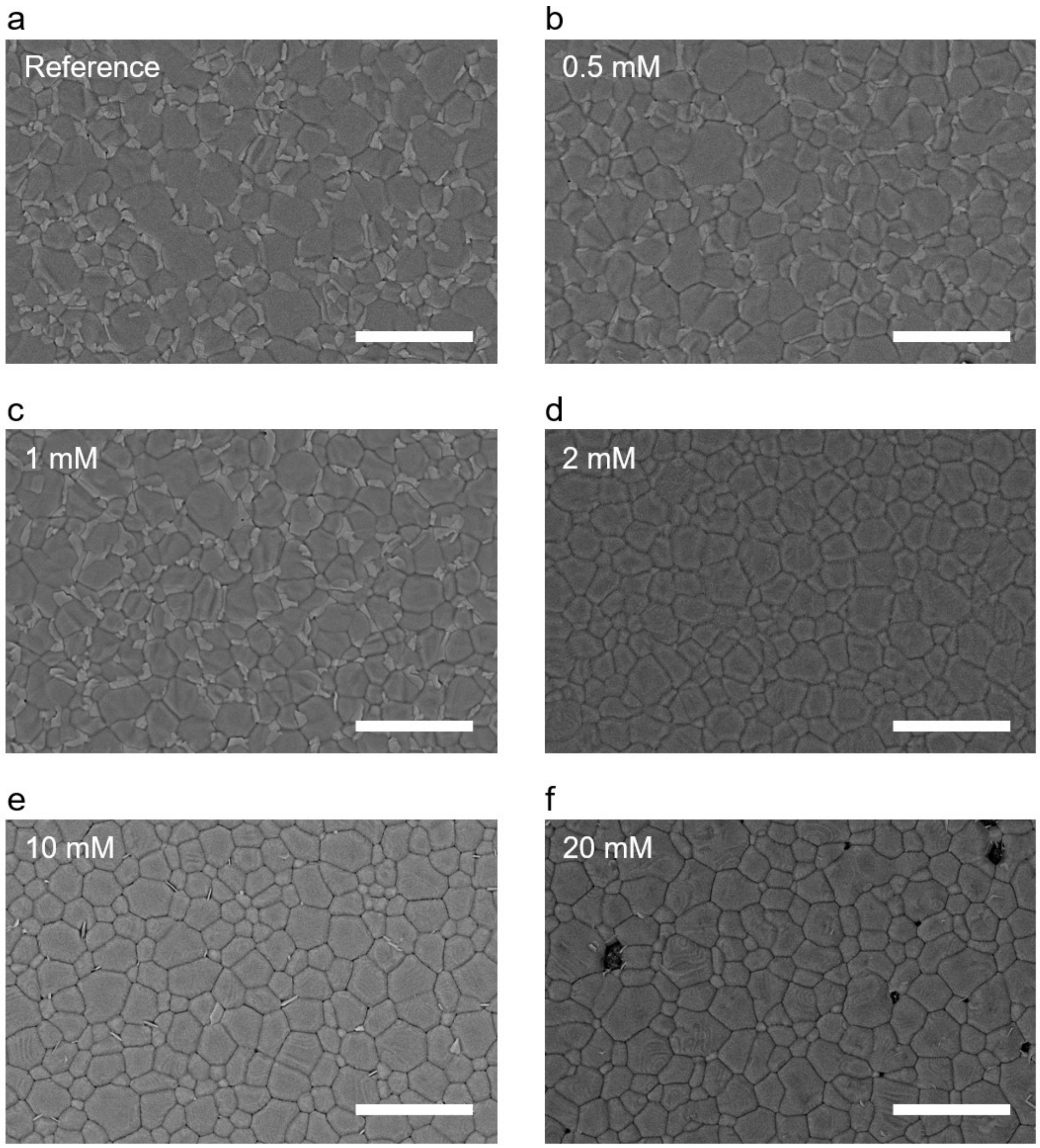

Fig. S2. Surface morphology of perovskite films. Scanning electron microscope surface images of **a,** reference**, b,** 0.5, **c,** 1, **d**, 2, **e,** 10 and **f,** 20 mM of [N4444][TFSI] used in the perovskite precursor solution**.** Scale bar is 5 μm.

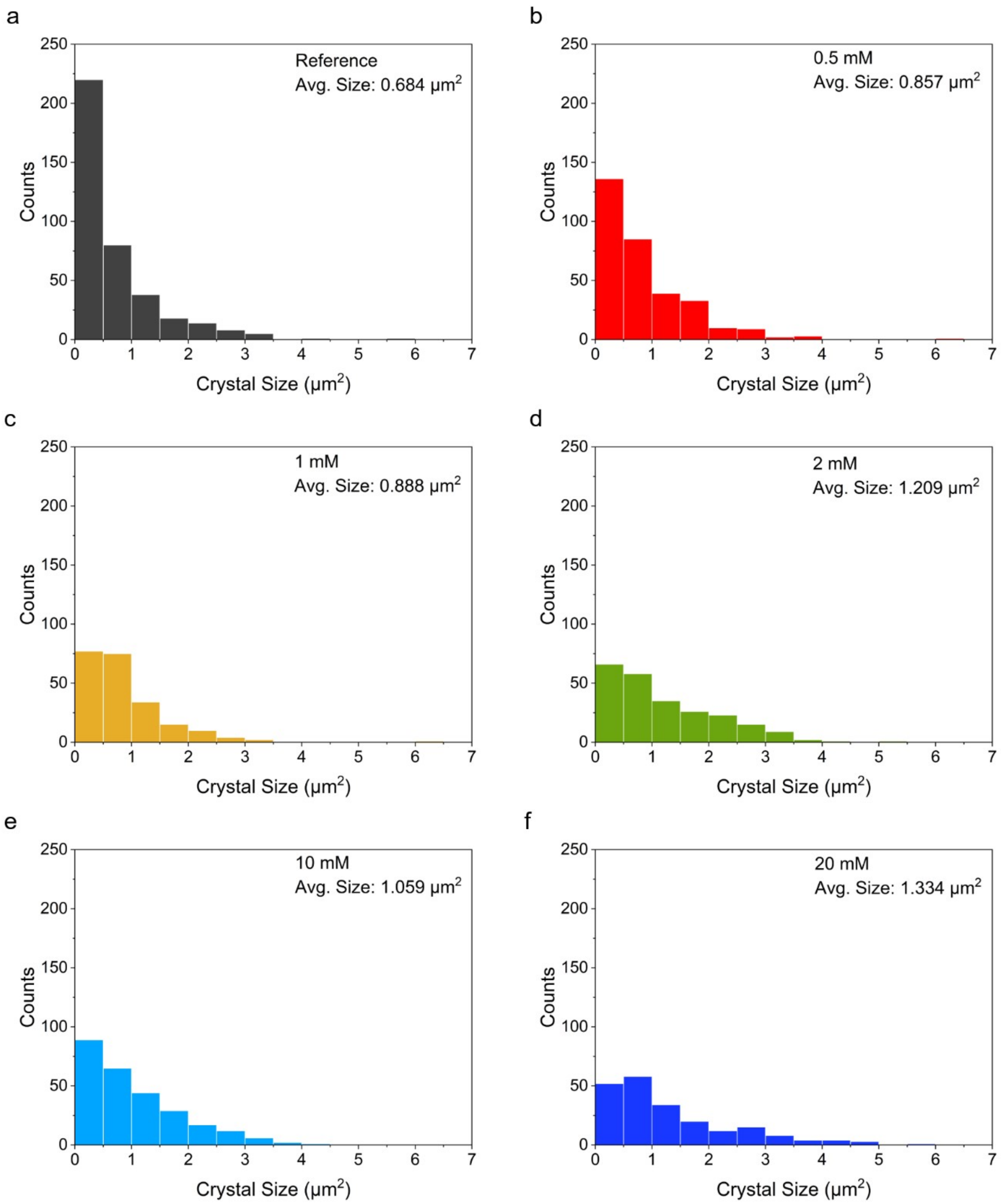

Fig. S3. Statistics data of crystal size for perovskite grains. Distribution of the perovskite crystal area regarding **a,** reference, **b,** 0.5 mM**, c,** 1 mM**, d,** 2 mM, **e,** 10 mM and **f,** 20 mM of [N4444][TFSI] used in the perovskite precursor solution.

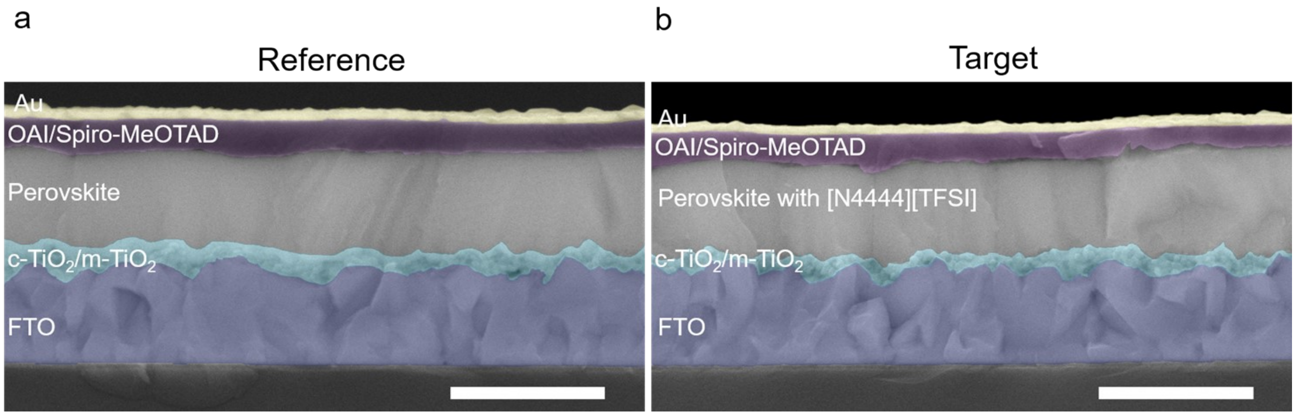

Fig. S4. Device structure. Cross-section SEM images of complete devices for **a,** reference and **b,** target (1 mM of [N4444][TFSI] used in the perovskite precursor solution). Scale bar is 1 μm.

Table S1. Data for cross-sectional thickness of reference and target samples from Fig. S4.

Thickness of reference perovskite film (nm)	Median thickness value of reference perovskite film (nm)	Average thickness value of reference perovskite film (nm)	Thickness of target perovskite film (nm)	Median thickness value of target perovskite film (nm)	Average thickness value of target perovskite film (nm)
573.222	606.6945	613.132	563.025	617.647	616.677
623.431			617.647		
560.669			571.429		
594.142			617.647		
602.510			626.050		
560.669			626.050		
589.958			579.832		
598.326			600.840		
594.142			588.235		
535.565			605.042		
569.038			630.252		
602.510			567.227		
665.272			588.235		
669.456			579.832		
602.510			621.849		
673.640			626.050		
623.431			575.630		
644.351			546.218		
610.879			592.437		
623.431			621.849		
652.72			651.261		
648.536			705.882		
610.879			634.454		
594.142			697.479		
686.192			710.084		
631.799			689.076		

Table S2. Data of fitting parameters for the time-resolved photoluminescence measurements of the perovskite films without and with different concentrations of [N4444][TFSI].

	A1	τ1 [μs]	A2	τ2 [μs]
Reference	0.8971	59.8	0.1046	438.6
0.5 mM	0.5375	403.4	0.3636	1768.8
1 mM	0.5405	456.8	0.3673	2085.0
2 mM	0.4624	566.6	0.4626	2551.7
5 mM	0.5154	653.0	0.4281	2931.2
10 mM	0.6592	615.7	0.2585	3658.7
20 mM	0.4981	677.2	0.3335	3936.8

†The equation for the fitting is $y=y_0+A_1\times exp(-(x-x_0)/\tau_a)+A_2\times exp(-(x-x_0)/ \tau_b)$.

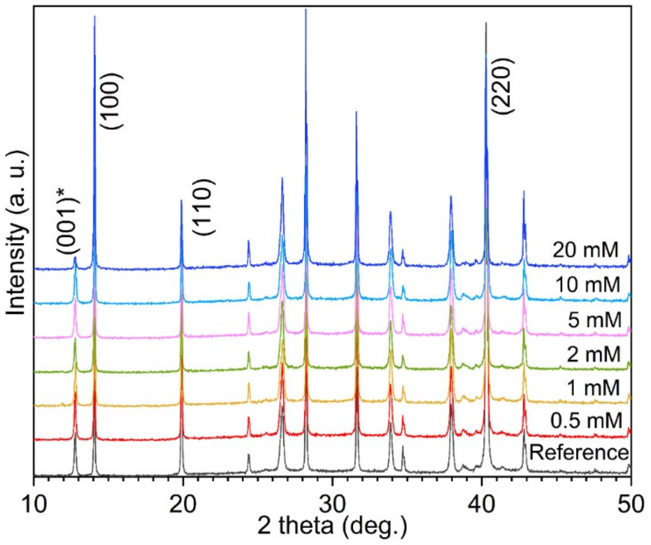

Fig. S5. XRD patterns of perovskite films. X-ray diffraction (XRD) of reference, 0.5, 1, 2, 10 and 20 mM of [N4444][TFSI] used in the perovskite precursor solution.

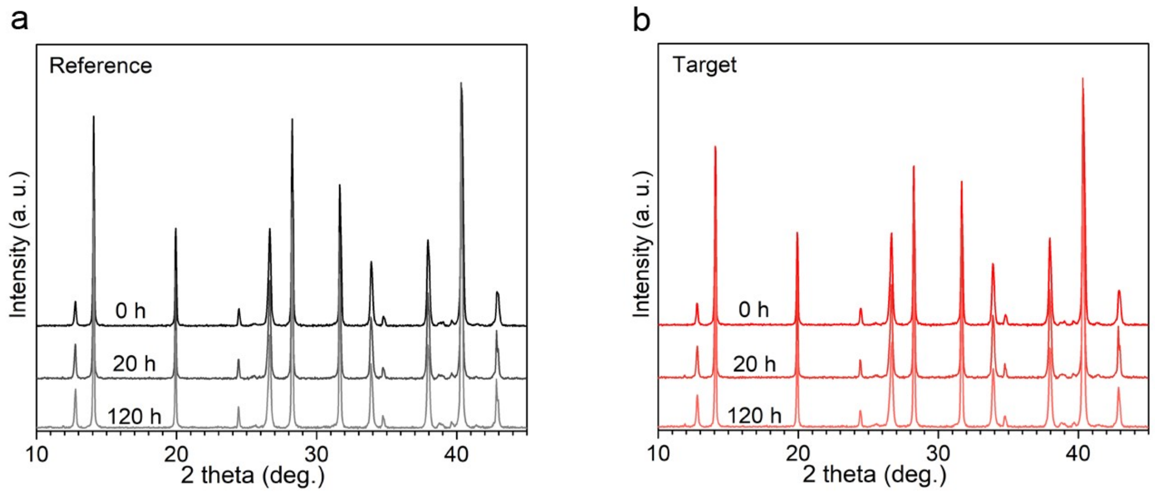

Fig. S6. XRD patterns of perovskite films as a function of time. a, XRD patterns of the reference and **b,** target exposed to 50% relative humidity.

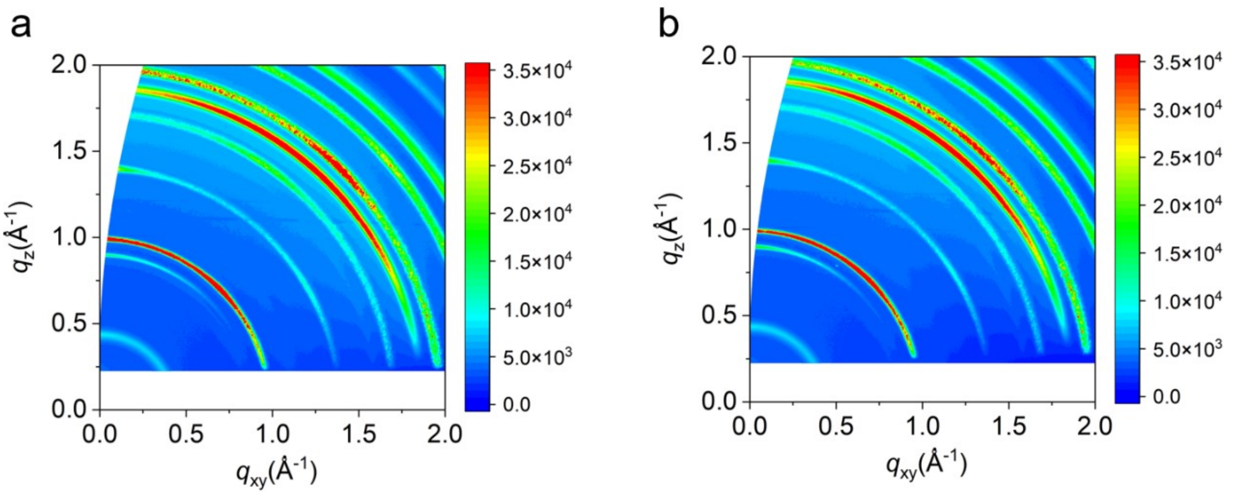

Fig. S7. Crystallinity of the perovskite film with and without [N4444][TFSI]. 2D-WAXS patterns of **a**, reference, **b**, 1 mM of [N4444][TFSI] used in the perovskite precursor solution.

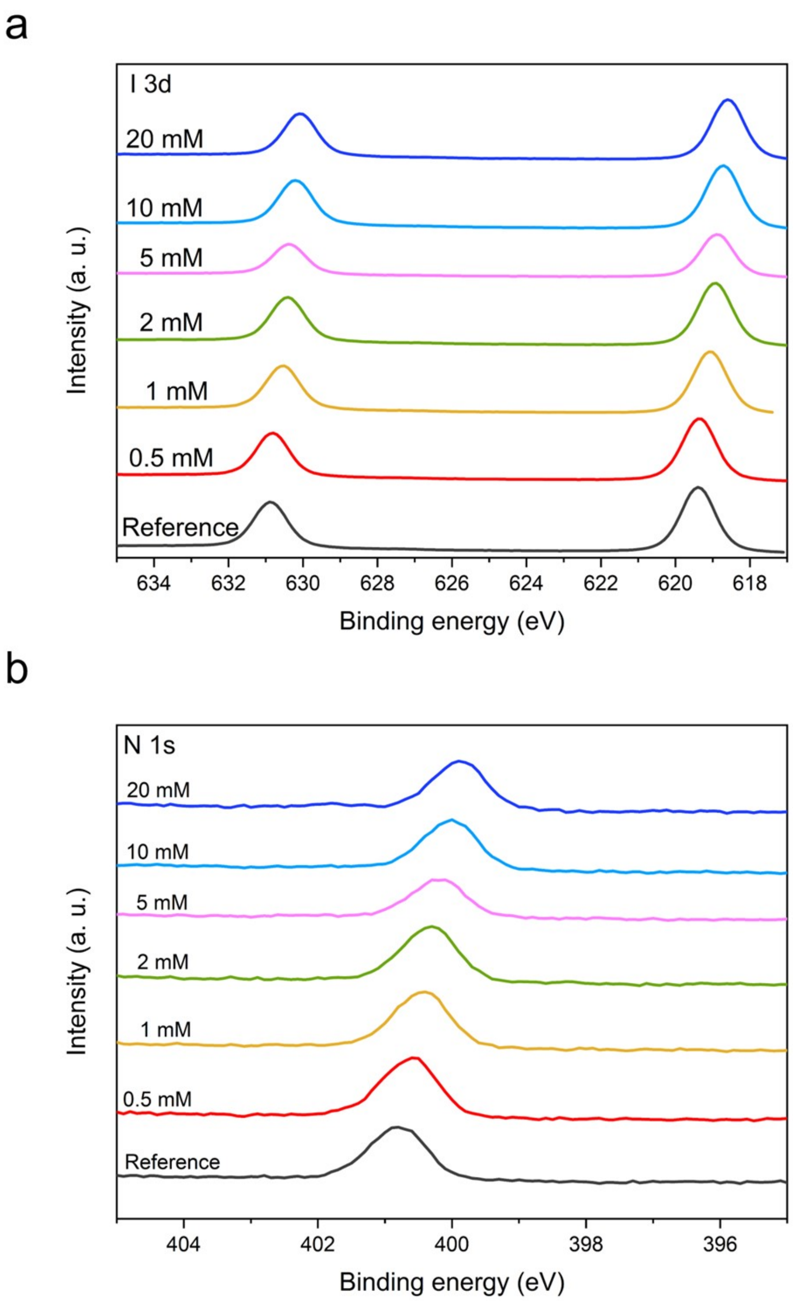

Fig. S8. Surface analysis of the perovskite films. X-ray photoelectron spectroscopy (XPS) regarding **a,** N 4s and **b,** I 3d of reference, 0.5, 1, 2, 10 and f, 20 mM of [N4444][TFSI] used in the perovskite precursor solution.

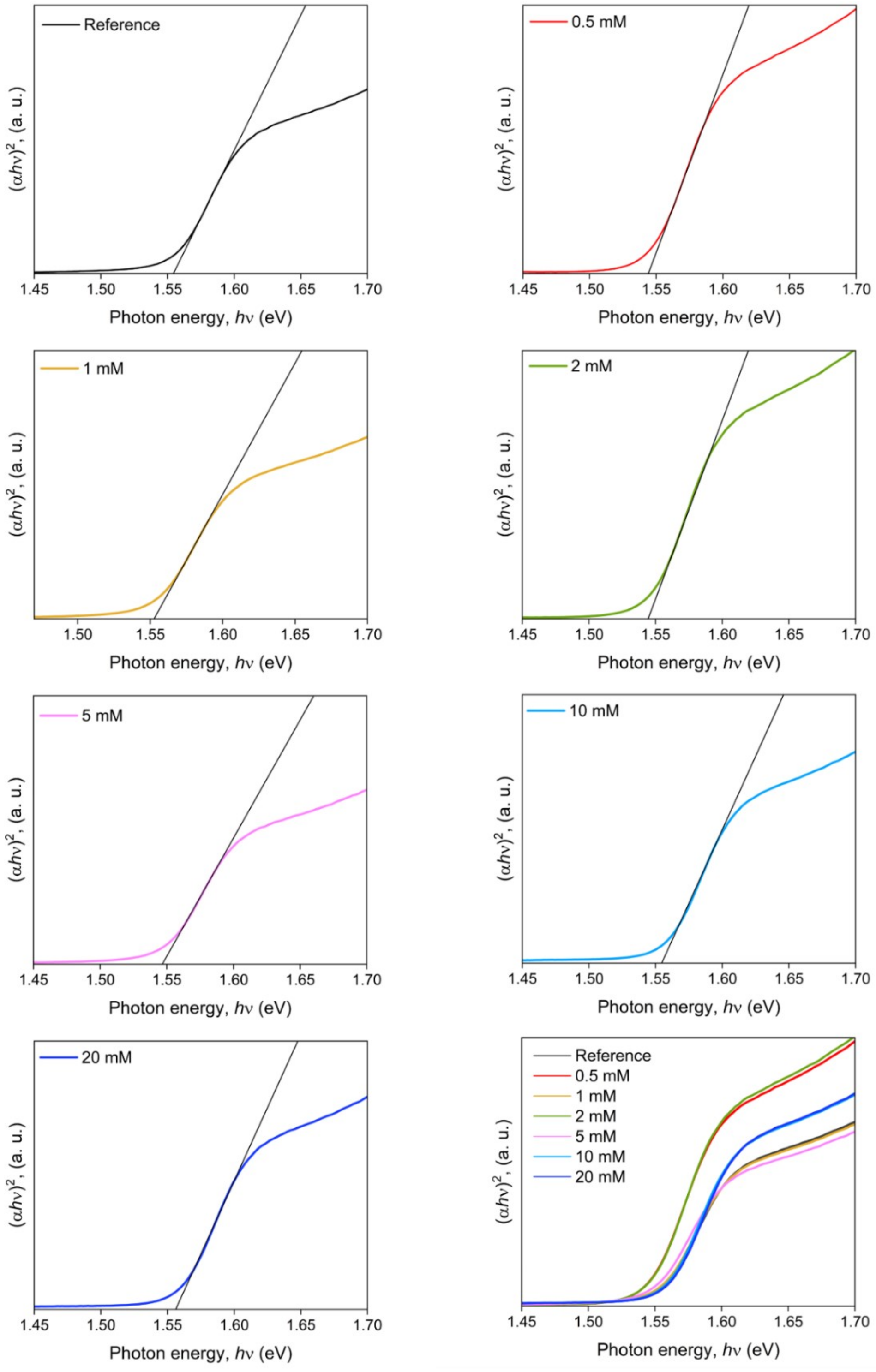

Fig. S9. Optical characteristics of the perovskite films. Tauc plots of the perovskite films regarding reference, 0.5, 1, 2, 5, 10, 20 mM of [N4444][TFSI] used in the perovskite precursor solution.

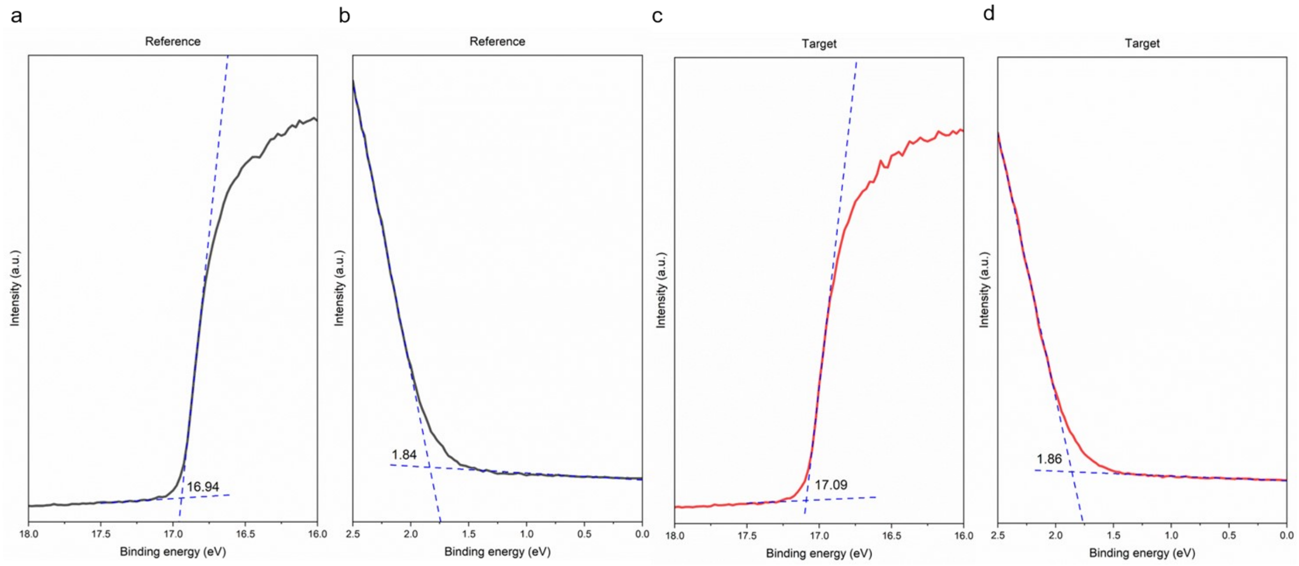

Fig. S10. Energy-level diagram. Secondary cut-off (a and c) and onset (b and d) from Ultraviolet photoelectron spectroscopy (UPS) of reference and target (1 mM of [N4444][TFSI] in perovskite precursor).

He-I source (21.22 eV) was used for UPS. In order to calculate HOMO (or valence band), photon energy (21.22 eV), secondary cut-off values (16.94 eV for reference, 17.09 eV for target) and onset (1.84 eV for reference, 1.86 eV for target) were utilized. For LUMO (or conduction band), HOMO values and band gap values were used.

Valence band of reference: 21.22 – (16.94 – 1.84) = 6.12 eV

Valence band of target: 21.22 – (17.09 – 1.86) = 5.99 eV

Conduction band of reference: 6.12 – 1.55 = 4.57 eV

Conduction band of target: 5.99 – 1.54 = 4.45 eV

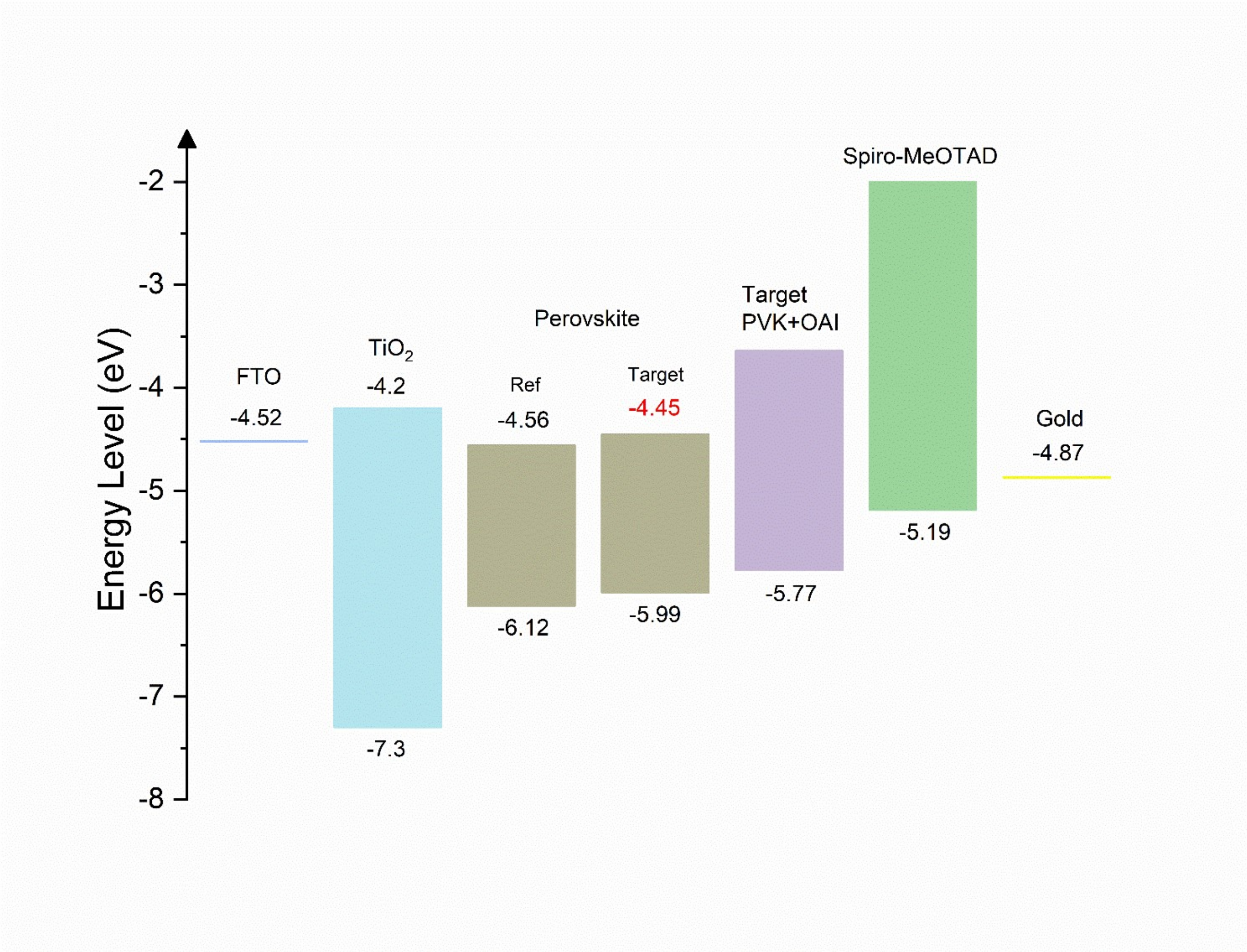

Fig. S11. Energy-level diagram. the energy level of devices structure (FTO/TiO_2/Perovskite (reference and target)/Perovskite+OAI)/Spiro-MeOTAD/Gold). The target is 1 mM of [N4444][TFSI] used in the perovskite precursor solution.

Table S3. Bandgap values of reference, 0.5, 1, 2, 10 and 20 mM of [N4444][TFSI] used in the perovskite precursor solution.

	**Reference**	**0.5 mM**	**1 mM**	**2 mM**	**5 mM**	**10 mM**	**20 mM**
**Bandgap (eV)**	1.554	1.544	1.553	1.545	1.547	1.555	1.556

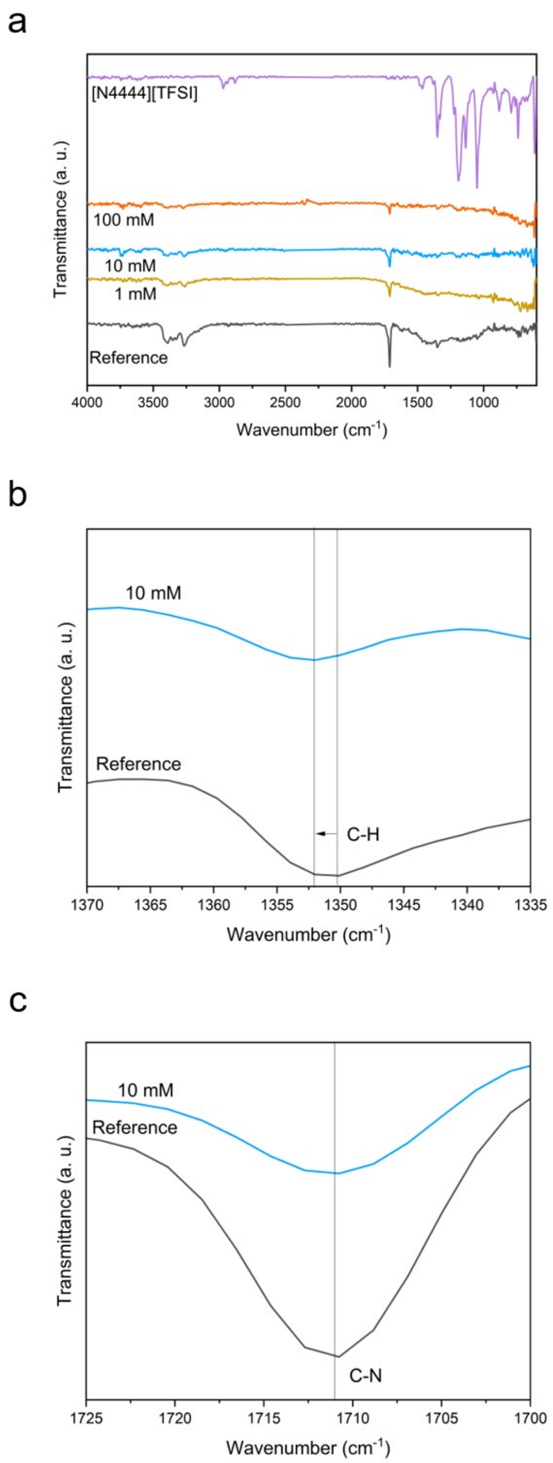

Fig. S12. Fourier-transform infrared spectroscopy (FTIR). FTIR spectra of **a,** reference, 10 mM and [N4444][TFSI] used in the perovskite precursor solution, **b**, C-H bending peak, **c**, C-N antisymmetric stretching peak of reference, 10 mM of [N4444][TFSI] used in the perovskite precursor solution.

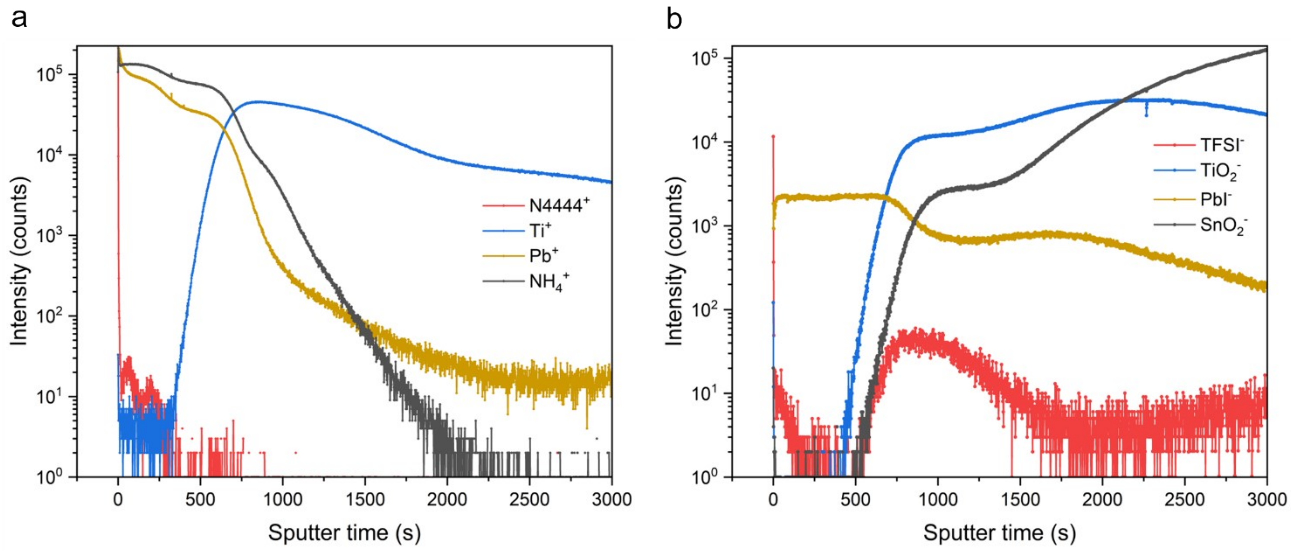

Fig. S13. Time-of-Flight Secondary Ion Mass Spectrometry (ToF-SIMS) depth profiling. The target sample structure (FTO/c-TiO_2/meso-TiO_2/$CsFAPbI_3$ with 1mM [N4444][TFSI].

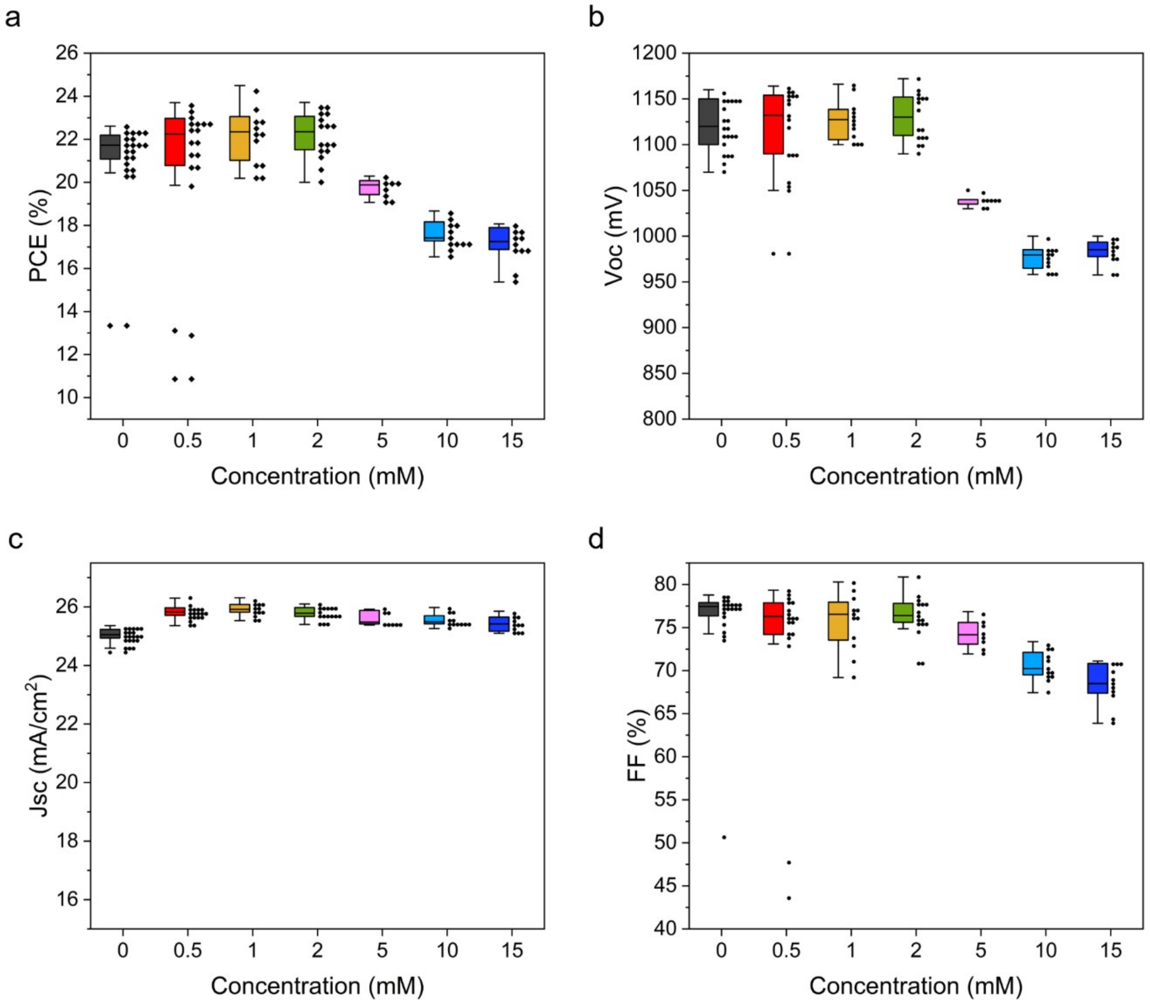

Fig. S14. Statistical photovoltaic data of perovskite solar cells. Box plots of reference (0), 0.5, 1, 2, 10 and 20 mM of [N4444][TFSI] used in the perovskite precursor solution. **a,** power conversion efficiency (PCE), **b,** open circuit voltage (V_{oc}), **c,** short circuit current density (J_{sc}), and **d,** fill factor (FF).

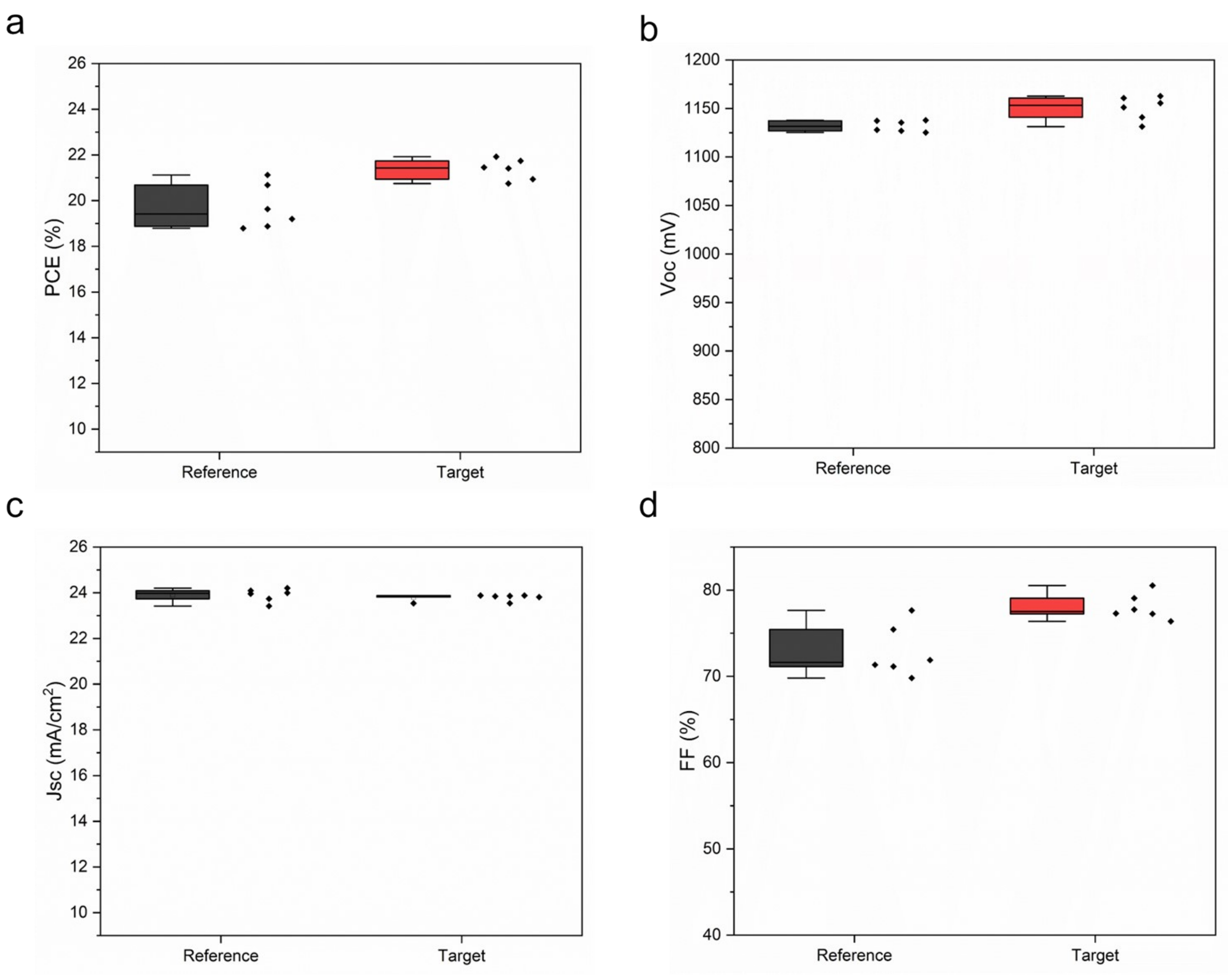

Fig. S15. Statistical photovoltaic data of bromine-doped perovskite solar cells (≈1.6 eV). Box plots of reference (0) and target (1 mM) of [N4444][TFSI] used in the perovskite precursor solution. **a,** power conversion efficiency (PCE), **b,** open circuit voltage (V_{oc}), **c,** short circuit current density (J_{sc}), and **d,** fill factor (FF).

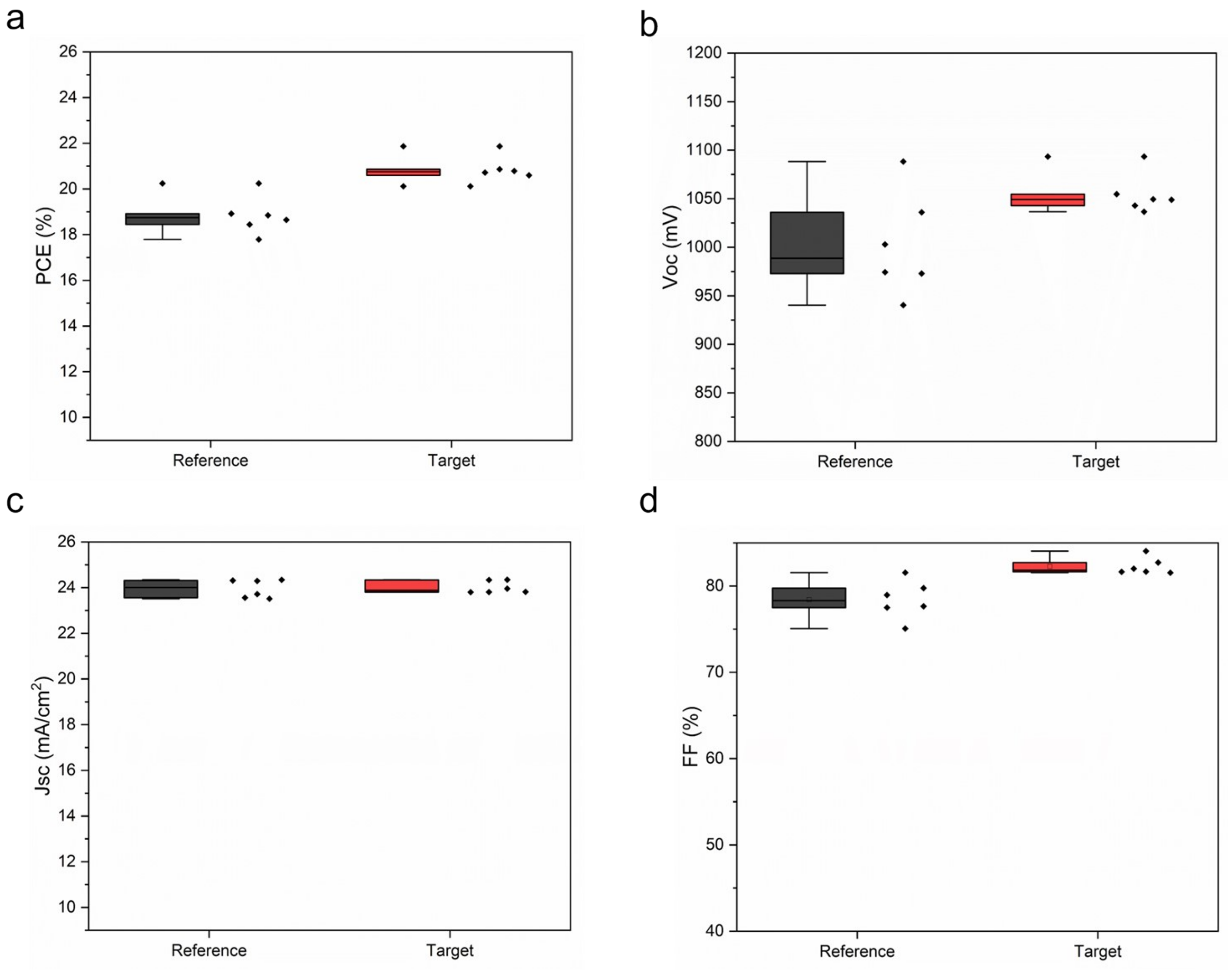

Fig. S16. Statistical photovoltaic data of perovskite solar cells regarding inverted structure. Box plots of reference (0 mM) and target (1 mM) of [N4444][TFSI] used in the perovskite precursor solution. **a,** power conversion efficiency (PCE), **b,** open circuit voltage (V_{oc}), **c,** short circuit current density (J_{sc}), and **d,** fill factor (FF).

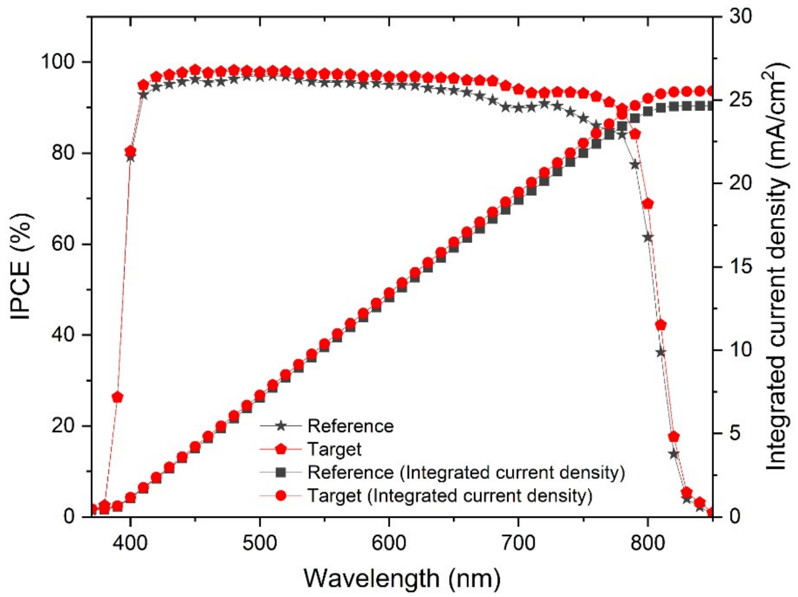

Fig. S17. Photovoltaic properties. Incident photon to electron conversion efficiency (IPCE) measurements and integrated current density data of reference (black colour) and target device (red colour).

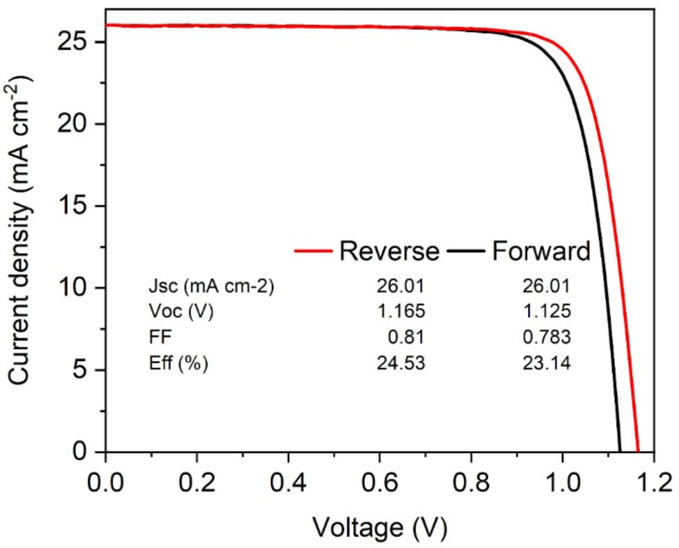

Fig. S18. Photovoltaic performances for hysteresis. Reverse and forward JV curves of champion target device.

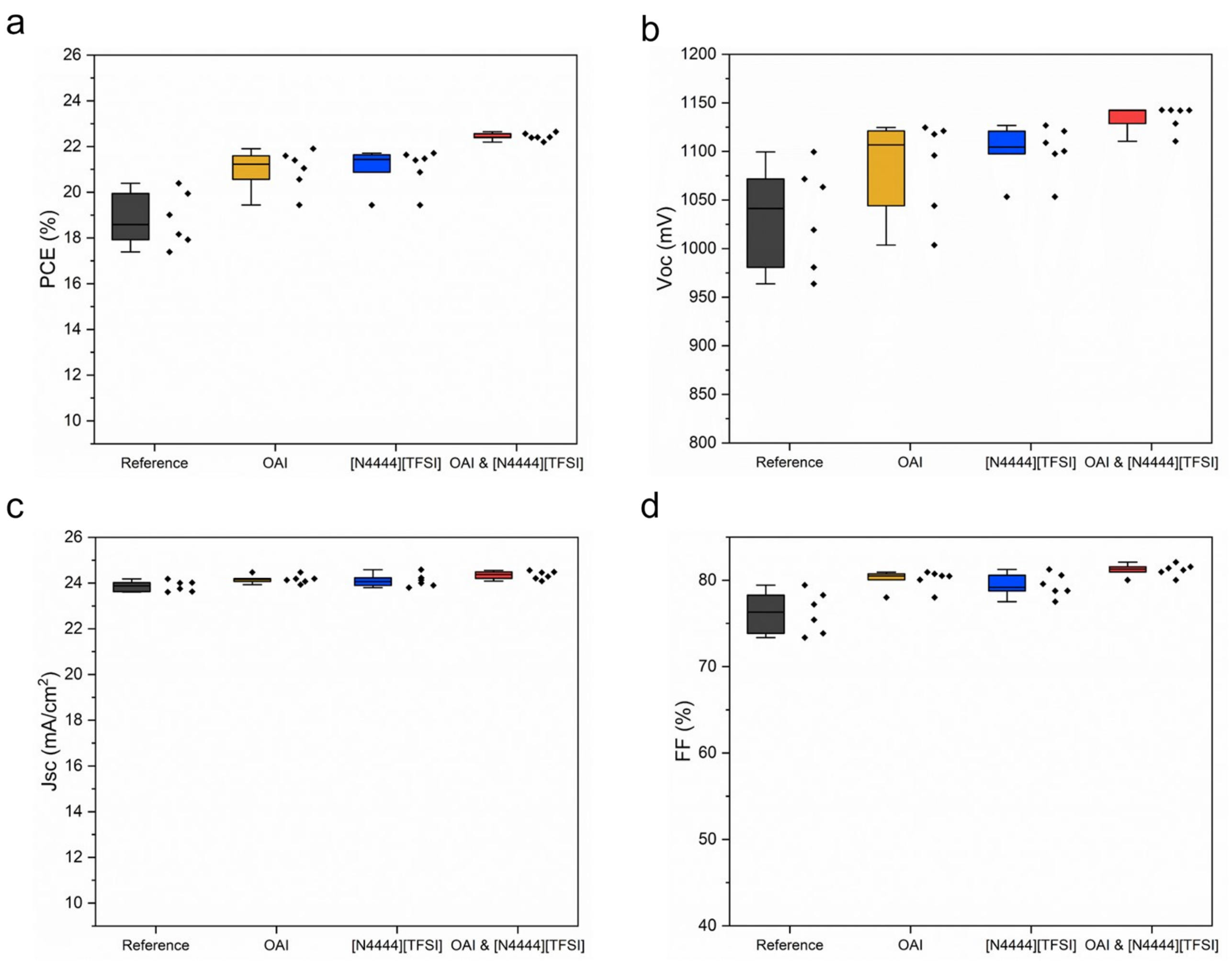

Fig. S19. Statistical photovoltaic data of perovskite solar cells in terms of [N4444][TFSI] additive and OAI. Box plots of reference (without [N4444][TFSI] and OAI), without [N4444][TFSI] and with OAI, with [N4444][TFSI] and without OAI, and target (with [N4444][TFSI] and with OAI). **a,** power conversion efficiency (PCE), **b,** open circuit voltage (*V*oc), **c,** short circuit current density (*J*sc), and **d,** fill factor (FF). Device structured <FTO/ETL/Perovskite/[N4444][TFSI]] passivation layer/OAI passivation layer/HTM/Au>

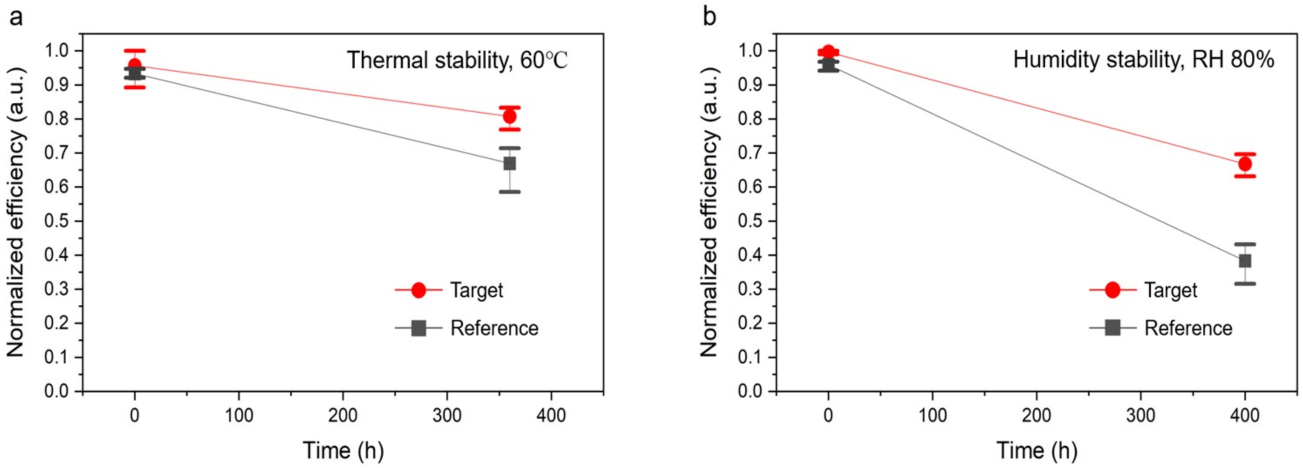

Fig. S20. Statistics data. (a) thermal stability (60°C) and (b) humidity stability (80% relative humidity) comparing reference and target devices. The number of samples was three devices for each condition. Each plot shows maximum, minimum, and average normalized efficiency.

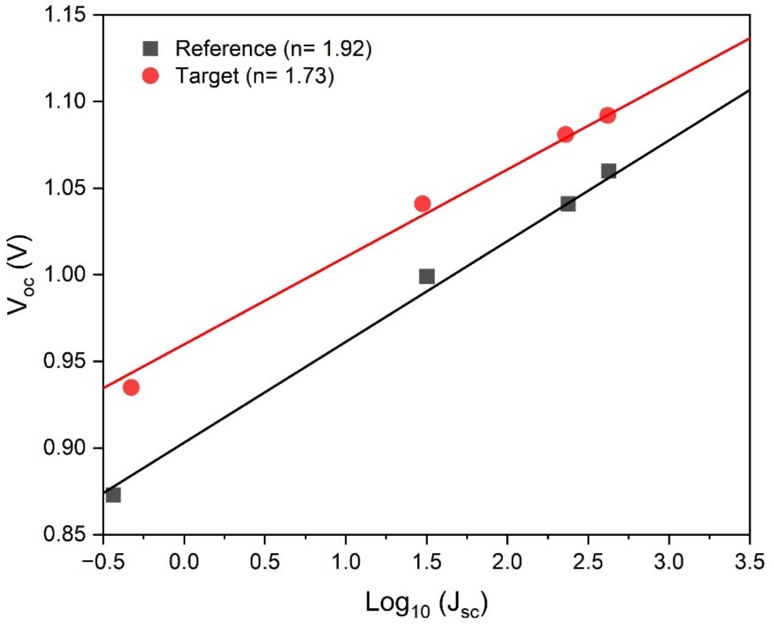

Fig. S21. Photovoltaic characteristics. Suns-Voc graph of reference and target perovskite solar cells.

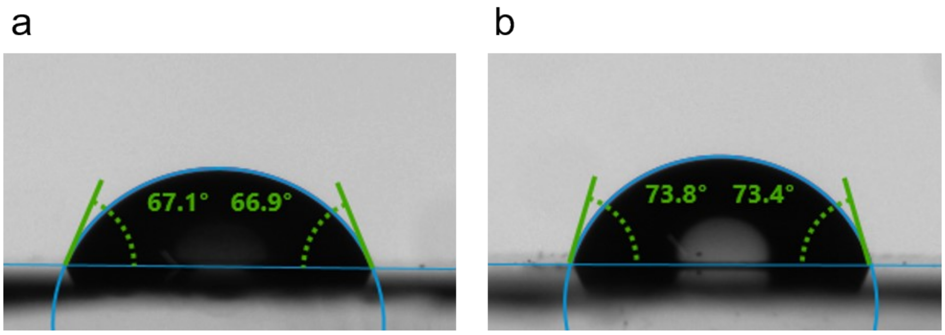

Fig. S22. Contact angle with a water droplet on the reference and target perovskite films. a, reference (average contact angle = 67.0°) and **b,** target perovskite film (average contact angle = 73.6°).
